# Identification of the Contamination Sources by PCBs Using Multivariate Analyses: The Case Study of the Annaba Bay (Algeria) Basin

**DOI:** 10.3390/molecules28196841

**Published:** 2023-09-28

**Authors:** Soumeya Khaled-Khodja, Hassen Cheraitia, Karima Rouibah, Hana Ferkous, Gaël Durand, Semia Cherif, Gamal A. El-Hiti, Krishna Kumar Yadav, Alessandro Erto, Yacine Benguerba

**Affiliations:** 1 Physical Chemistry of Materials Laboratory, Faculty of Sciences and Technology, Chadli Bendjedid University, BP 73, El Tarf 36000, Algeria; s.khaled-khodja@univ-eltarf.dz; 2 Department of Mathematics, Faculty of exact sciences, Jijel University, BP 98, Ouled Aissa, Jijel 18000, Algeria; hassen.stat@hotmail.fr; 3 Laboratory of Materials: Elaborations-Properties-Applications LMEPA, Jijel University, BP 98, Ouled Aissa, Jijel 18000, Algeria; karima.rouibah@univ-jijel.dz; 4 Département de Chimie, Faculté des Sciences, Université de 20 Août 1955 de Skikda, Skikda 21000, Algeria; h.ferkous@univ-skikda.dz; 5 Laboratoire de Génie Mécanique et Matériaux, Faculté de Technologie, Université de 20 Août 1955 de Skikda, Skikda 21000, Algeria; 6 Public Laboratory Expertise and Analysis Consulting in Bretagne, C.S. 10052, 29280 Plouzané, France; gael.durand@labocea.fr; 7 Materials and Environment Research Laboratory for Sustainable Development LR18ES10, ISSBAT, Tunis University El Manar, Tunis 1006, Tunisia; semia.cherif@issbat.utm.tn; 8 Department of Optometry, College of Applied Medical Sciences, King Saud University, Riyadh 11433, Saudi Arabia; gelhiti@ksu.edu.sa; 9 Faculty of Science and Technology, Madhyanchal Professional University, Ratibad, Bhopal 462044, India; envirokrishna@gmail.com; 10 Environmental and Atmospheric Sciences Research Group, Scientific Research Center, Al-Ayen University, Nasiriyah 64001, Thi-Qar, Iraq; 11 Dipartimento di Ingegneria Chimica, dei Materiali e della Produzione Industriale, Università Di Napoli Federico II, 80125 Napoli, Italy; 12 Laboratoire de Biopharmacie et Pharmaco Technie (LBPT), Department of Process Engineering, Faculty of Technology, Ferhat ABBAS Setif-1 University, Setif 19000, Algeria; yacinebenguerba@univ-setif.dz

**Keywords:** urban waste, wastewater, POPs, PCB contamination sources, PCA/HCA, multivariate analysis

## Abstract

Persistent Organic Pollutants (POPs), particularly the indicator polychlorinated biphenyls (PCBs), were first quantified in water and sediments of two wadis, Boujemaâ and Seybouse, as well as in the effluents from a fertilizer and phytosanitary production industrial plant (Fertial). Since these contaminated discharges end in Annaba Bay (Algeria) in the Mediterranean Sea, with a significant level of contamination, all the potential sources should be identified. In this work, this task is conducted by a multivariate analysis. Liquid–liquid extraction and gas chromatography/mass spectrometry (GC–MS) methods were applied to quantify seven PCB congeners, usually taken as indicators of contamination. The sum of the PCB concentrations in the sediments ranged from 1 to 6.4 μg/kg dw (dry weight) and up to 0.027 μg/L in waters. Principal component analysis (PCA) and hierarchical cluster analysis (HCA) were used for the multivariate analysis, indicating that the main sources of PCB emissions in the bay are urban/domestic and agricultural/industrial. The outfalls that mostly contribute to the pollution of the gulf are the Boujemaâ wadi, followed by the Seybouse wadi, and finally by the Fertial cluster and more precisely the annex basin of the plant. Although referring to a specific site of local importance, the work aims to present a procedure and a methodological analysis that can be potentially applicable to further case studies all over the world.

## 1. Introduction

Persistent organic pollutants (POPs) encompass a diverse group of organic compounds known for their toxic properties and remarkable resistance to natural environmental degradation processes. These pollutants have a propensity to persist and accumulate through biomagnification in the food chain, making them a significant environmental concern [1]. Among the various POPs, organochlorine compounds, such as polychlorinated biphenyls (PCBs), pose a particularly severe threat due to their persistence and high toxicity [2,3,4,5].

PCBs are complex mixtures of synthetic organochlorine compounds sharing a similar chemical structure but differing in the positions and numbers of chlorine atoms. Indicator PCBs (PCBi), consisting of seven congeners (PCB 28, 52, 101, 118, 138, 153, and 180), are commonly quantified in environmental matrices due to their persistence and toxicological impact [2,3,4,5].

These compounds are of great concern because of their high toxicity, resistance to degradation, carcinogenic properties, and long-range transport capabilities [6,7,8]. PCBs not only persist in the environment but also accumulate in the fatty tissues of organisms, posing health risks to both wildlife and humans. Exposure to PCBs can lead to dermal lesions, hepatotoxicity, immunosuppression, endocrine disruption, and neurotoxicity [9]. Consequently, in the early 1970s, the production and usage of PCBs were curtailed due to their identified hazards [1,4]. Recognizing their environmental and health risks, the Stockholm Convention, held in 2001, classified PCBs as priority pollutants and called for restrictions on their production and use to protect both human health and the environment [10,11].

The unique characteristics of PCBs, including their chemical stability and low water solubility, result in their accumulation in environmental sinks, such as soils and sediments, where they exhibit long half-lives [12,13,14,15,16,17,18]. PCBs were historically used in various industrial applications, including heat exchangers, dielectric fluids, pesticides, inks, paints, paper, and plastics, contributing to their widespread distribution in the environment [1,3,5,14,19,20]. Despite the ban on PCBs in most countries, including Algeria in 2001, their persistent nature has left a legacy of contamination in Algerian soils and waters, varying in levels based on the sources and activities in affected areas [21,22]. This ongoing contamination continues to affect aquatic organisms and poses a threat to human health through the food chain [23]. The accurate assessment and diagnosis of PCB-contaminated areas are crucial for minimizing human exposure and ensuring environmental and public health safety.

While the global concern for POPs is well-documented, there is a notable lack of comprehensive studies on PCBs in surface soils around Mediterranean cities in North Africa, especially in Algeria [4]. This knowledge gap is particularly relevant given Algeria’s significant size, with an area of 2,381,741 km^2^, making it the largest country in Africa, the Arab world, and the Mediterranean basin. Its extensive coastline, stretching over 1200 km along the Mediterranean Sea, faces increasing pressure due to diverse anthropogenic activities, with over 51% of the national industry located along this coastal fringe [24].

The industrialization process in Algeria, conducted without due consideration for ecological factors, has resulted in severe environmental challenges, including water pollution from wastewater discharge, air pollution from various emissions, and inadequate management of special waste, all of which threaten the environment and public health [24,25].

Annaba, an industrial and tourist city in northeastern Algeria, is emblematic of these environmental challenges. Its coastline, part of the Mediterranean, is highly contaminated due to decades of discharges of domestic, industrial, agricultural, and hospital wastewater, often untreated, into the Mediterranean Sea via numerous wadis [24,25,26,27,28]. This environmental predicament is not unique to Annaba but is characteristic of many Mediterranean coastal cities [29].

Despite the evident contamination, data on organic pollution in coastal catchments and freshwater ecosystems in Algeria are sparse, with limited information on PCB levels and their sources in various surface soils used for different purposes [4,30,31,32]. Additionally, the potential contamination of other aquatic systems, such as rivers acting as receivers of organic contaminants from municipal and industrial wastewater, remains poorly understood [33,34,35].

This study, conducted within the framework of a broader project on urban discharges in the Gulf of Annaba and their impact on the pelagic ecosystem, addresses this knowledge gap. Specifically, it aims to:

(i) Quantify the seven European indicators of PCBs in water and sediments from various sources contributing to discharges in the Gulf of Annaba, including the Fertial fertilizer complex, Wadi Boujemaâ, and Wadi Seybouse. (ii) Identify and differentiate the origins (agricultural, industrial, and domestic) of these contaminants at various stations using multivariate techniques, such as principal component analysis (PCA) and hierarchical cluster analysis (HCA) [36].

While the study is conducted at a specific local site, its methodology and findings can serve as a model for similar investigations worldwide, contributing to a broader understanding of PCB contamination and its sources. This research also aligns with the objectives set forth in the Libreville Declaration of 2008, emphasizing the importance of strategic health and environment alliances in Africa.

## 2. Results

In this section, we present the PCB concentrations found in the water and sediments of the various stations under observation. The results obtained in this assessment were compared with other results reported by other studies related to the same pollutant, but on different sites.

Subsequently, we aimed to identify the stations that contribute to the degradation of water and sediment in the Gulf basin the most and differentiate between the agricultural, industrial, and domestic origins of these contaminants by processing the data using two complementary statistical methods (PCA and HCA).

### 2.1. PCB Concentration in Water Samples

The seven PCBs analyzed in the water of the various stations were below the detection limit (less than 0.005 µg/L). Anx Fertial is the only station where three PCBs were found: PCB138, PCB153, and PCB180. Their concentrations fluctuate from 0.006 to 0.011 µg/L (Table 1).

The sum of PCB concentrations in Anx Fertial samples was 27 times higher than the limit value (0.001 μg/L) recommended by NQEp (norms of provisional environmental quality). It can be concluded that the water of this station is of very poor quality with regard to PCBs.

### 2.2. PCB Concentration in Sediments

All the PCB indicators were found in the sediments of all the stations, except PCBs 28 and 118. Their concentrations varied between 0.33 and 2.07 μg/kg dw (Table 2). The sum of the PCBs varied significantly from one station to another. The highest concentrations of PCBs were found in the Boujemaâ wadi and then the Seybouse wadi, followed by Anx Fertial and PB Fertial.

Boujemaâ wadi is distinguished from the other stations by a greater contribution of PCBs in terms of both number of congeners found and their concentration. Indeed, this wadi contains the largest number of PCBs with the highest concentrations.

Therefore, we can infer that the Boujemaâ wadi, which conveys domestic wastewater on the west side of the city of Annaba, is very rich in organic matter, favoring the adsorption of PCBs on solid sediment. It is the station that contributes to the pollution of the Annaba Gulf basin by PCBs the most. In the second position, the Seybouse wadi, which crosses many agricultural lands that extend widely on its banks, carries domestic discharges from several agglomerations. The industrial releases of Anx Fertial station rank third in contaminating coastal waters with PCBs. Finally, the cooling waters of the PB Fertial station, which initially are marine waters but after passing through the cooling circuit of the plant come out contaminated by PCBs, provide a minor contribution.

The PCB concentrations found in this assessment were compared with the results reported by other studies (Table 3) and referred to the same pollutants (and expressed as the sum of all the congeners) but in different sites. It appears that the Annaba Bay basin is more polluted by PCBs than the other industrial sites located in the northern and western parts of the country (Algeria). It is also more polluted than the bay of Monastir, Lake Ichkeul, and the lagoon of Bizerte, located in Tunisia. The Egyptian coast, western Niger Delta, Yisilirmak River in Turkey, and Galvaston Bay in Texas (USA) are less polluted than the Gulf of Annaba basin as well. However, the other sites for which data are available, located in Congo, France, Italy, Belgium, Lebanon, China, and the Black Sea in Turkey, are relatively more polluted than the Annaba Bay basin.

It is also distinguished from the other stations by a greater contribution of PCBs in terms of both number and concentration. Indeed, this wadi contains the largest number of PCBs with the highest concentrations.

We can therefore infer that the Boujemaâ wadi, which conveys domestic wastewater on the west side of the city of Annaba, is very rich in organic matter, favoring the adsorption of PCBs on solid sediment. It is the station that contributes to the pollution of the Annaba Gulf basin by PCBs the most. In the second position, the Seybouse wadi, which crosses considerable agricultural land that extends widely on its banks, carries domestic discharges from several agglomerations. The Anx Fertial station, whose discharges are purely industrial, ranks third in the contamination of coastal waters by PCBs. Finally, the cooling waters of the PB Fertial station, which originally are marine waters but after passing through the cooling circuit of the plant come out contaminated by PCBs, provided the least contribution.

## 3. Discussion

The concentration of the seven PCBi in the water at the various stations remains below the detection limit (0.005 μg/L), except for the Anx Fertial station (Table 1). The latter is mainly contaminated by PCB138, PCB153, and PCB180, whose concentrations far exceed the limit (0.001 μg/L) recommended by NQEp. This excess of PCBs can be attributed to the fact that Anx Fertial receives liquid waste from the manufacturing processes of phytosanitary products.

Of the seven PCBi investigated in the sediments of the various stations, five were identified. Their concentrations varied significantly from one station to another (Table 2). However, the highest concentrations were recorded in the Boujemaâ wadi (Urban/Industrial area), followed by the Seybouse wadi (Urban/Industrial/Agricultural area), Anx Fertial, and PB Fertial (Industrial areas).

Wastewater from the western side of the city of Annaba is discharged into the Boujemaâ wadi. Its clay substrate, rich in organic matter, encourages the retention of these pollutants in the substrate, hence the high rates. The relatively high levels of PCBs in the Seybouse wadi can be explained by the fact that it covers a long distance (240 km) and receives domestic and agricultural wastewater from more than 86 municipalities, before discharging into coastal waters. The Anx Fertial station shows more PCBi than the PB Fertial station. This can be explained by the fact that the Anx Fertial plant receives wastewater directly from the manufacturing processes of phytosanitary products and other chemicals, whereas the PB Fertial plant receives cooling water from the Fertial complex.

A further and deeper analysis can be conducted by applying the PCA method to identify the degree of pollution in the Annaba bay basin and determining the PCBs responsible for the water quality degradation of the Annaba Bay basin. Moreover, the HCA methodology allows the separation of the different stations into distinct clusters according to the type of discharge, the nature of the sediments, and the contribution to the contamination of the bay basin.

### 3.1. Principal Components Analysis and Hierarchical Clustering Analysis (PCA/HCA)

To establish the typology of the various stations regarding the sediments, the PCA and HCA tools were applied to the retrieved dataset. Since the PCB concentrations in the waters of the various stations were almost all below the detection limit, we applied multivariate analyses (PCA/HCA) only to the sediments where the PCBs were found. PCBs whose concentrations were below the detection limits were not taken into consideration either.

#### 3.1.1. PCA Applied to PCB Concentrations Found in Sediments

The PCA processing of the results for PCB concentrations found in sediments from the various stations showed that the cumulative contributions of the first two axes (Dim 1 and Dim 2) fully explain the variance (100%).

**Figure 1:**
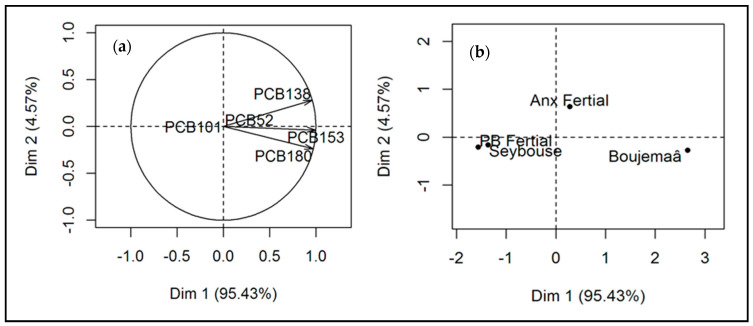
PCA applied on PCBs (variables) (**a**) and individuals (stations) (**b**) in sediments from various stations (First axis: Dim 1 and Second axis: Dim 2).

The eigenvalues were 2.86, 0.14, and 0.00 respectively for the three axes (Dim 1, Dim 2, and Dim 3). Their respective contributions to the total variance were 95.43%, 4.57%, and 0.001%. As the first two axes explained 100% of the variance, they were used for the factor analysis (Figure 1).

According to the factorial plane (Dim 1 × Dim 2) of the PCA (Figure 1a), we observed that axis 1 (Dim 1) summarizes the essential information. The inertia rates were around 95% for Dim 1 and around 5% for Dim 2.

The congeneric PCBs that are well represented on Dim 1 and strongly contribute to its construction were PCB138, PCB153, and PCB 180. The latter evolve in the same positive direction, which may reflect their common origin, while PCB101 is different to the other PCBs concerning axis 1, which may reflect its different origin relative to the other three PCBs. Indeed, PCB 101 was found only in the Boujemaâ wadi.

This typology of variables (PCBs) corresponds to a typology of records (stations), which allows us to identify the dominant trends. The observation chart (Figure 1b) shows that the four stations PB Fertial, Anx Fertial, Boujemaâ, and Seybouse are independent regarding the sources of water pollution by PCBs. It also shows that the contributors to the construction of axis 1 are mainly the Boujemaâ and the Seybouse wadis and the PB Fertial. However, the Boujemaâ wadi is different to the Seybouse and the PB Fertial. This disjunction can be explained by the nature of the substrate and the type of release. The Boujemaâ wadi, with its clayey substrate and very rich organic matter, better retains micropollutants, unlike the Seybouse wadi and the PB Fertial station, which have sandy sediments. This also explains the higher levels of PCBs in the Boujemaâ samples, followed by the Seybouse and PB Fertial samples.

In addition, discharges into the Boujemaâ wadi are mainly urban and residential, whereas the discharges from the Seybouse are mainly agricultural and industrial. This last origin, therefore, associates it with the effluents from PB Fertial, which are of the industrial type.

Axis 1 seems to reflect the presence of PCBs from various sources, mainly domestic, agricultural, and industrial.

Anx Fertial is the only station that is well represented by axis 2. Knowing that this station carries wastewater from the manufacturing process of fertilizers, phytosanitary products, and detergents, it can be stated that axis 2 reflects the exclusively industrial origin of the PCBs coming from this station.

#### 3.1.2. HCA Applied to PCB Concentrations Found in Sediments

**Figure 2:**
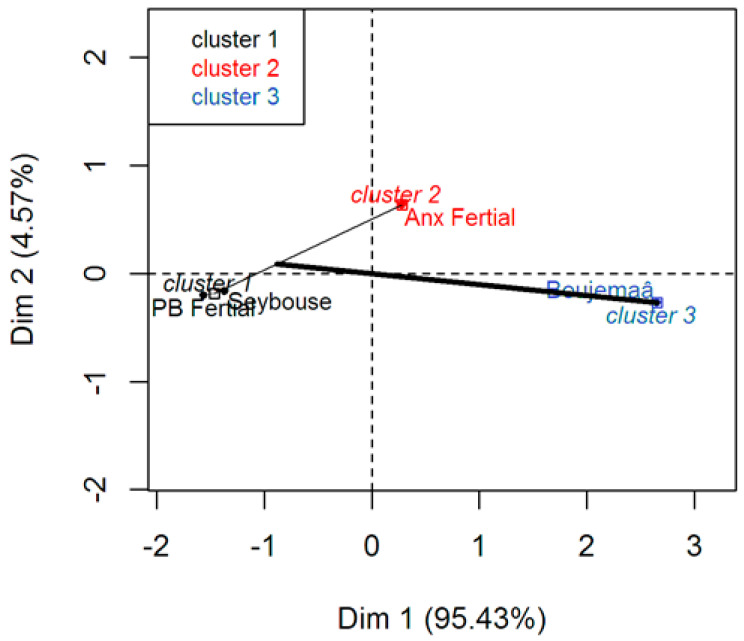
Distribution of the stations and clusters (axes F1 × F2).

The hierarchical cluster analysis (HCA) (Figure 2) identified three separate clusters: the first is formed by PB Fertial and the Seybouse wadi, the second cluster is formed by Anx Fertial, and finally the last cluster is represented by the Boujemaâ wadi.

Cluster 1 formed by Seybouse wadi and Station PB Fertial highlights the similarities that exist between these two stations. First, the nature of the substrate of both stations is sandy. Second, the Seybouse wadi station is located at the mouth (interface), i.e., the water of the Seybouse is in direct contact with the coastal waters. These are pumped by the Fertial complex to cool the plant and then discharged back into the bay via the PB Fertial station.

Cluster 2 is represented by Anx Fertial. This station is distinguished from the other stations by its purely industrial wastewater discharges.

Cluster 3 is represented by the Boujemaâ wadi.

Finally, we noted that the distances between the different clusters are relatively large. This may highlight the diversity of the sources of contamination and the nature of the discharges.

According to the first axis (Dim 1), the PCBs that contribute to pollution in the Gulf basin the most derive from the Urban/Industrial effluents of Cluster 3, while the effluents that contribute the least to contamination are the Agricultural/Urban/Industrial effluents from Cluster 1.

Axis 2 (Dim 2) represents only effluents of industrial origin (Cluster 2), which also contribute to the contamination of the Gulf basin, but to a lesser extent than the Cluster 3 effluents.

## 4. Materials and Methods

### 4.1. Presentation and Location of the Study Sites and Stations in Annaba Gulf

**Figure 3:**
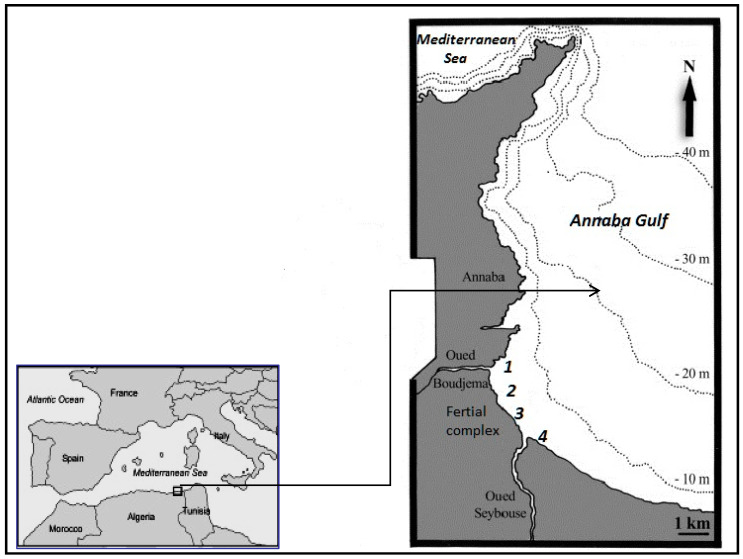
Location of study sites and stations in Annaba Gulf (1. Boujemaâ wadi, 2. Anx Fertial, 3. PB Fertial, 4. Seybouse wadi).

The Gulf of Annaba is located in the extreme east of Algeria (36°50′–37° N; 7°45′–8°15′ E) (Figure 3). In the Bay of Annaba, the modified Atlantic current moves eastwards from the seaward side and crosses the Annaba shelf, which allows some renewal of the outer neritic waters. However, the inner part of the gulf is mainly influenced by the continental inputs of the Seybouse and Boujemaâ wadis. The urban waste of the more than a million inhabitants of the city of Annaba and the surrounding villages, as well as the industrial waste from the Fertial fertilizer manufacturing complex, are discharged into the coastal waters without prior treatment.

The selected sites under study are the main sources of known anthropic discharges in the Annaba Bay. In particular, the site of the Fertial industry complex (Figure 3), whose main business is the manufacture of ammonia, phosphate fertilizers, nitrogen fertilizers, ammonium nitrate fertilizers, complex fertilizers, phytosanitary products, and detergents, contributes significantly to the discharges.

Located on the seaside and about 4 km from the town of Annaba (Algeria), the complex is bordered to the north by the Mediterranean Sea, to the south-west by the city of El Bouni, to the south-east by El Hadjar and the municipality of Sidi Salem, and to the northwest by the town of Annaba. Fertial is the source of many environmental and health problems (such as air pollution and respiratory diseases) through its liquid and gaseous discharges. Two representative stations of the complex were chosen. The first station is the main basin, where the seawater used to cool the plant is discharged, referred to as PB Fertial (3) (Figure 3). The second station is a smaller basin, where all the wastewater from the manufacture of fertilizers, detergents, and phytosanitary products is collected, referred to as Anx Fertial (2) (Figure 3). Both basins discharge their liquid effluents directly into coastal waters.

The second site is Boujemaâ wadi (1) (Figure 3), which collects the largest urban effluent of Annaba city. It runs 15 km through the western plain of the city of Annaba and draws from its swamps during the rainy season. It is also the last tributary of the Seybouse wadi. It collects the major wastewater entering the bay, crosses the west plain of Annaba city, and ends up in the sea. Before reaching the sea, the effluent receives considerable domestic sewage from several sewer lift stations and many other connections from domestic sewage. It is estimated that the effluent carries the domestic waste of more than 100,000 inhabitants. In addition, it receives minor effluents from the Fertial complex and discharges from the slaughterhouse located near the wadi [31,32]. It is therefore one of the major sources of wastewater input to the Gulf of Annaba.

Not including riverine water, the effluent has a flow varying generally along the day from 0.2 to 1 m^3^/s. It is ultimately a real wastewater outfall [27].

The third site is the Seybouse wadi (4) (Figure 3) situated in the north-eastern end of Algeria. This wadi is ranked second in the country for its vast drainage basin (6471 km^2^) and length. However, it is considered one of the most polluted wadis in Algeria [24] as many anthropic discharges (urban, agricultural, and industrial effluents) converge to the sea. In fact, multiple anthropogenic activities take place at the low plain of Seybouse (e.g., heavy industries, the national company of wood, the regional company of cement, industrial groups of paper and cellulose, the company of regional manufacturing of medical articles, and the complex of Fertial). Given that its land is viewed as the most fertile in Algeria, varied forms of agriculture occupy the whole of the lower plain of Seybouse: cattle, truck farming, industrial crops (tobacco), forage crops, cereal, and fruit cultivations are located in the region [52,53]. The wadi, 240 km long, crosses more than 86 municipalities in which domestic wastewater is discharged without pretreatment, due to the absence of treatment plants [54]. The various discarded waters join at the Seybouse mouth before reaching the coast. Finally, both Boujemaâ and Seybouse wadis are situated near a road with intense traffic, and there is also a gas station next to Seybouse wadi, which contribute as further sources of contamination.

### 4.2. Sampling

To estimate the concentrations of PCBs in waters and sediments, a seasonal sampling was collected during the years of 2009–2010: a campaign in October 2009 (autumn period), another one in February 2010 (winter period), a third one in May 2010 (spring period), and a last one in August 2010 (summer period).

Water samples for PCBs analysis were collected from each site in 1 L pre-cleaned glass amber bottles, with Teflon-lined screw caps [55]. They were manually collected from surface waters, no deeper than 1 m. All sampling vessels were pre-cleaned with acetone, deionized water, and washed three times with the sampled water.

Surface sediments (0–5 cm) were collected with a stainless-steel scoop [56], at the same places where the water samples were taken. The samples for the determination of PCBs were stored in glass vials washed with a detergent (Decon, East Sussex, UK), rinsed with ultrapure water and acetone, and finally dried at 120 °C before use.

After sampling, the water and sediments were transported in a cool box at 4 °C to the laboratory LABOCEA (Bretagne Oceane, France), where they were stored at −20 °C until analysis.

### 4.3. PCB Extraction and Analysis

#### 4.3.1. Water Samples

PCBs were liquid–liquid extracted with a hexane/dichloromethane mixture (50:50, *v*/*v*) [57]. After the separation, the organic extract was evaporated down to 2–4 mL, and the micropollutants were separated with chromatography on activated silica gel. The fractions were concentrated under a vacuum and stored before analysis.

#### 4.3.2. Sediment Samples

Sediment samples were dried at 40 °C for 24 h, ground, and sieved at 2 mm. The sieved samples (10–25 g), spiked with internal standards, were extracted by a Soxhlet system for 24 h by 300 mL of n-hexane for PCB analysis. The extracts were concentrated and purified by liquid chromatography on a silica gel column. PCBs were recovered by elution with 30 mL of a diethyl ether/hexane mixture (1:10, *v*/*v*). The fractions were concentrated using a rotary evaporator followed by a flow of nitrogen. Internal standards were added before quantification by instrumental analysis [58].

The separation, identification, and quantification of PCBs in water and sediments were performed using gas chromatography coupled with mass spectrometry (7890 gas chromatograph (GC) coupled with a 5975C mass selective detector (MSD), Agilent Technologies Inc., Palo Alto, CA, USA). The system is based on a chromatographic separation based on retention time and peak intensity, coupled with compound identification based on molecule ionization, which breaks them into fragments. The mass of the resulting fragments is specific for each molecule and can be used to identify the chemical. The MS was operated in the selected ion-monitoring (SIM) mode. The mass spectra were compared to the reference libraries of known compounds to be identified.

#### 4.3.3. Data Analysis

The standards used for the assessment of water and sediment quality of freshwater ecosystems were based on the French system of evaluation of stream water quality (SEQ-Eau) recommended by French water agencies [55] and the circular of 7 May 2007, DCE/23 defining the standards of provisional environmental quality (NQEp) for French surface waters [59].

For the marine environment, the references chosen to determine the quality of the water are the NQEp. The quality of marine sediments was assessed using two grids: the grid established by the Study and Observation Group on Dredging and the Environment GEODE (French interministerial decree of 14 June 2000, J.O.10.80) and the assessment grid for the level of chemical contamination of the French coastline provided by IFREMER’s French national observation network.

To identify the sources of PCB contamination in the Annaba Bay basin and to explore the possible relationships between all the stations studied, two different statistical techniques were applied to the results obtained: principal component analysis and hierarchical clustering analysis (PCA/HCA).

PCA and HCA are the most widely used tools to explore similarities and hidden patterns among samples where the relationship between data and clusters is unclear [60,61]. PCA is a multivariate analytical tool widely used for receptor modeling in environmental pollution source apportionment studies [62]. It is an exploratory data analysis technique used to reduce the complexity of data sets to their most important components. It describes the relationships that exist between several variables simultaneously and transforms correlated variables into new, uncorrelated variables called ‘principal components’ (PCs), ‘principal axes’, or ‘principal factors’, enabling grouping of data with similar behaviors [63]. Furthermore, it allows the practitioner to reduce the number of variables and make the information less redundant [60,64]. It also provides a graphical representation that leads interpreting more easily the results, which are generally restricted to the first two factorial designs (PC1 and PC2), provided that these explain most of the variance in the cloud of initial variables [60,64].

HCA is a statistical clustering method that explores the organization of samples into groups and among groups depicting a hierarchy. Clusters in the data are identified based on similarities between samples. Usually, samples are grouped by calculating the Euclidean distance between them, followed by a stepwise grouping of the most similar variables. The result of HCA is presented in a dendrogram, a plot that shows the organization of samples and their relationships in tree form. The shortest distances between branches represent the greatest similarities [14,64].

HCA is a complementary technique of PCA. The combination of these statistical techniques is considered useful for obtaining information about the existing site clusters with similar pollution characteristics and for identifying the sources of contamination [48,63]. Specifically, the PCA enabled us to identify the PCBs that contribute most to the contamination of the Gulf basin. The principal components (axes: Dim 1 and Dim 2) showed that water quality in the basin is mainly controlled by domestic wastewater effluents and industrial discharges.

HCA revealed the various sources of contamination in the bay basin. On the basis of water quality, the four sampling stations were grouped into 3 clusters: less polluted sites (industrial area), moderately polluted sites (agricultural/industrial area) and heavily polluted sites (domestic/residential area). Statistical analyses were performed using the software R-3.4.3. program for Windows with ‘R.utils’ package.

## 5. Conclusions

This study aimed at individuating and characterizing the contamination of waters (fresh and marine) and sediments of the regions located in the southwest of the Mediterranean and more particularly at the northeast end of Algeria. Telluric discharges of all kinds (domestic, agricultural, and industrial) were discharged into the wadis, without prior treatment, and inevitably ended up in Annaba Bay. This situation leads to the degradation of freshwater resources, the shortage of which has already been felt for several years. The pollution of the most productive coastal waters can have disastrous consequences on fishery resources and public health through the consumption of contaminated seafood. In particular, the study focused on the individuation of PCBs in four chosen stations and represents one of the few studies realized about these major contaminants.

The analysis carried out clearly showed that despite the use of PCBs has been banned for years in Algeria, these micropollutants are still present in the environment.

The PCB concentrations in the waters of the various stations remain below the detection limit except for the discharges from Anx Fertial where the PCB concentrations greatly exceed the limit recommended (0.001 mg/L according to NQEp) and thus conferred poor quality to the water. Five congeneric PCBs, deriving from various sources, were detected in the sediments of all the stations. The sediment of the Boujemaâ wadi contains the greatest number of PCBs with the highest concentrations, coming most probably from urban and residential sources. The second position resulted in the Seybouse wadi, whose number of PCBs is equivalent to that of the Boujemaâ wadi but the concentrations remain much lower. The source of these PCBs seems to be related to agricultural discharges and, to a lesser extent, industrial and domestic discharges. PCBs of exclusively industrial origin are found in the Anx Fertial station, which holds the third position with a relatively low number of PCBs, but concentrations equivalent to those of Seybouse. PB Fertial resulted in the last with the lowest number and concentrations of PCBs.

From a quality point of view, the sediments from Boujemaâ and the Seybouse wadis seem to be within the limits recommended by the SEQ-Eau for the freshwater stations, and within the standards of the RNO and the GEODE, for the marine stations (PB Fertial and Anx Fertial).

However, it should not be forgotten that we took superficial sediments and that the sandy substrate of the Seybouse wadi and the two basins of Fertial can promote the infiltration of these POPs, thereby obtaining these low concentrations.

Finally, the PCA tool applied to the data set enabled us to distinguish the different sources of contamination, which are in the following descending order: urban (domestic)/residential sources, agricultural/industrial sources, and industrial sources. The HCA allowed us to define the stations that most contribute to the degradation of the waters of the Gulf basin: the Boujemaâ wadi, the Seybouse wadi, and the Fertial complex.

The presence of PCBs in the environment, even in low concentrations, constitutes a real threat to aquatic ecosystems and public health. This is why it is imperative to carry out systematic monitoring of these xenobiotics in water and sediments to control their release into the natural environment and prevent their bioconcentration in the food chain [65].

## Figures and Tables

**Table 1:** Individual concentrations of PCBs (µg/L) in the water samples collected in four sampling stations.

Compounds		Stations		
	Boujemaâ	Seybouse	PB Fertial	Anx Fertial
PCB_28_	<0.005	<0.005	<0.005	<0.005
PCB_52_	<0.005	<0.005	<0.005	<0.005
PCB_101_	<0.005	<0.005	<0.005	<0.005
PCB_118_	<0.005	<0.005	<0.005	<0.005
PCB_138_	<0.005	<0.005	<0.005	0.006
PCB_153_	<0.005	<0.005	<0.005	0.01
PCB_180_	<0.005	<0.005	<0.005	0.011
∑PCBs	<0.005	<0.005	<0.005	0.027

Explanation: ∑PCBs: sum of polychlorinated biphenyl concentrations; Boujemaâ: downstream of the Boujemaâ wadi station; Seybouse: Seybouse wadi mouth station; PB Fertial: principal basin of the Fertial complex; Anx Fertial: the second basin of the Fertial complex.

**Table 2:** PCB concentration in sediments (µg/kg dw).

Compounds	Stations			
	Boujemaâ	Seybouse	PB Fertial	Anx Fertial
PCB 28	<0.9	<0.9	<0.9	<0.9
PCB 52	<0.6	0.6	<0.6	<0.6
PCB 101	0.7	<0.6	<0.6	<0.6
PCB 118	<0.9	<0.9	<0.9	<0.9
PCB 138	1.6	0.47	0.27	0.42
PCB 153	2.07	0.6	0.4	0.6
PCB 180	2.03	0.37	0.33	0.58
∑PCBs	6.4	2.04	1	1.6

**Table 3:** Concentration levels of PBCs in Annaba Bay basin and those found in other sites, with an indication of the different types of sampling areas (agricultural, urban/residential, and industrial).

Location Site	Type of Sampling Area	∑PCBs μg/kg	References
Basin Bay/Annaba	Urban/Industrial/Agricultural	1–6.4	This study
Industrial sites in northern Algeria	Industrial	0.001–0.128	[21]
East of Oran City/Algeria	Urban/Residential	0.002–0.019	[4]
Arzew/Algeria	Industrial	n.d.–0.015	[4]
Monastir Bay/Tunisia	Urban	0.001–0.008	[37]
Ichkeul Lake–Bizerte Lagoon Complex/Tunisia	Industrial/Agricultural	0–0.01	[38]
Mediterranean coast/Egypt	Industrial/Agricultural/Urban	0.20–1.92	[39]
Makelele River/Congo	Urban/Industrial	22.14–169.29	[40]
Atlantic Coastal/Congo	Urban/Industrial/Agricultural	12.06–2013	[41]
Western Niger Delta/Nigeria	Urban/Industrial	0.98–6.34	[42]
Berre Lagoon/France	Agricultural/Urban/Industrial	468.84–541.4	[43]
Huveaune River/France	Industrial/Agricultural	2.8–435	[44]
Western Scheldt River/Belgium	Urban/Industrial	105–400	[45]
Ionian Sea/Southern Italy	Industrial/Urban	2–1648	[46]
Gulf of Taranto/Italy	Industrial	85–1780	[47]
Gulf of Naples/Italy	Urban/Industrial/Agricultural	3.9–253	[48]
Coastal Zone/Lebanon	Urban/Industrial	143–303	[2]
Black Sea/Turkey	Industrial	1.03–23.72	[49]
Yisilirmak River/Turkey	Urban/Agricultural	0.7–1.15	[49]
Sanya River/China	Urban/	1.75–92.75	[14]
Laizhou Bay/China	Urban/Industrial	0.68–6.56	[50]
Galveston Bay/Texas	Industrial	0.004–0.1	[51]

Explanation: n.d.: Not detected.

## Data Availability

Not applicable.
